# Facile Synthesis of Nanoporous Pt-Y alloy with Enhanced Electrocatalytic Activity and Durability

**DOI:** 10.1038/srep41826

**Published:** 2017-02-02

**Authors:** Rongjing Cui, Ling Mei, Guangjie Han, Jiyun Chen, Genhua Zhang, Ying Quan, Ning Gu, Lei Zhang, Yong Fang, Bin Qian, Xuefan Jiang, Zhida Han

**Affiliations:** 1 Department of Chemistry and Materials Engineering, Changshu Institute of Technology, Changshu 215500, People’s Republic of China; 2 Department of Chemistry and Materials, Hebei Normal University, Shijiazhuang 050024, People’s Republic of China; 3 Jiangsu Laboratory of Advanced Functional Materials, Department of Physics, Changshu Institute of Technology, Changshu 215500, People’s Republic of China; 4 School of Materials Science and Engineering, China University of Mining & Technology, Xuzhou 221116, People’s Republic of China; 5 Suzhou Key Laboratory of Food Quality and Safety, School of Biology and Food Engineering, Changshu Institute of Technology, Changshu 215500, People’s Republic of China

## Abstract

Recently, Pt-Y alloy has displayed an excellent electrocatalytic activity for oxygen reduction reaction (ORR), and is regarded as a promising cathode catalyst for fuel cells. However, the bulk production of nanoscaled Pt-Y alloy with outstanding catalytic performance remains a great challenge. Here, we address the challenge through a simple dealloying method to synthesize nanoporous Pt-Y alloy (NP-PtY) with a typical ligament size of ~5 nm. By combining the intrinsic superior electrocatalytic activity of Pt-Y alloy with the special nanoporous structure, the NP-PtY bimetallic catalyst presents higher activity for ORR and ethanol oxidation reaction, and better electrocatalytic stability than the commercial Pt/C catalyst and nanoporous Pt alloy. The as-made NP-PtY holds great application potential as a promising electrocatalyst in proton exchange membrane fuel cells due to the advantages of facile preparation and excellent catalytic performance.

Proton exchange membrane fuel cells (PEMFCs), which convert the hydrogen and oxygen directly into electricity and water, are expected to play a key role in a future society based on sustainable energy^1^,^2^. A major challenge for the wide commercialization of PEMFCs lies in the low natural abundance of Pt and the slow oxygen reduction reaction (ORR) at the cathode^3^,^4^,^5^,^6^,^7^. Therefore, it is critical to develop novel materials with superior ORR electrocatalytic activity and higher durability in comparison with Pt to reduce or replace the Pt loading in the cathode. Although alloying Pt with transition metals (such as Fe, Co, Ni, Cu, etc) can effectively enhance both the catalytic activity and utilization of Pt^8^,^9^,^10^,^11^,^12^,^13^,^14^,^15^,^16^,^17^,^18^,^19^,^20^,^21^, usually their long-term performance is weakened by their poor stability resulting from dealloying under corrosive circumstance^22^,^23^,^24^,^25^. Recently, Huang *et al*. achieved both activity and stability in surface dopped Pt_3_Ni octahedral supported on carbon by transition metals, providing a promising way to enhance the stability by surface engineering^20^. Nevertheless, further improvements could be engendered by developing novel Pt-based alloy catalysts with enhanced activity and durability under operating conditions.

Recent theoretical calculations and experimental results have demonstrated that alloys of Pt and rare earths, such as Y, La, Ce, Gd, Tb, Dy, are both active and stable as electrocatalysts for ORR^26^,^27^,^28^,^29^,^30^. Among them, Pt-Y alloys have attacted much attention, including Pt_3_Y^26^ and Pt_5_Y^31^. The superior ORR performance of Pt-Y alloys were confirmed experimentally in the form of polycrystalline surfaces and thin films^26^,^31^,^32^,^33^,^34^. Chorkendorff and co-workers demonstrated that the Pt-Y nanoparticles prepared through the gas-aggregation technique exhibited high ORR activity ever measured on an extended polycrystalline surface^35^. Most recently, electrodeposition in anhydrous metal salt solutions, such as ionic liquids and alcohol solution, has been proved to be an effective way to prepare nanostructured Pt-Y alloy^36^,^37^. However, the above synthesis methods of Pt-Y catalysts may be cost-consuming and still complicated, which do not allow for mass production. In order to maximize the ORR activities of Pt-Y alloys, it is necessary to develop the chemical synthesis of high-quality nanostructured Pt-Y catalysts in bulk quantities, in a simple and low-cost way. Several researchers have attempted to chemically synthesize nanostructured Pt-Y or similar catalysts^38^,^39^,^40^, but the prepared materials don’t show superior catalytic performance in the ORR. This may due to high affinity of rare-earth elements for oxygen which inhibits the formation of Pt-Y alloy. Thus, a method for mass production of nanosized Pt-Y alloy as promising electrocatalyst in PEMFCs is strongly desired but remains a significant challenge.

Dealloying, the selective dissolution of an active component in appropriate corrosion conditions from a alloy, has been proved to be an effective way to generate three-dimensional bicontinuous nanoporous metals^41^,^42^,^43^. A typical commercial application is Raney nickel prepared by chemical dealloying of NiAl_3_, which has been used as a heterogeneous catalyst for more than 80 years^44^. As-prepared nanoporous materials have received great attention because a unique combination of a highly conductive network and a highly accessible open nanoporosity is favorable for the electron and mass transportation during electrocatalysis^45^. Recnently, various nanoporous alloys of Pt and late transition metals (LTM), such as Fe, Ni, and Cu, were successfully synthesized by dealloying method and exhibited improved ORR activity than pure Pt^46^,^47^,^48^. In contrast, the case of nanoporous Pt-Y alloys, is more challenging and has not been reported up to now, because Y has a much higher negative reduction potential than LTM. These features inspire us to explore: (1) whether nanoporous Pt-Y alloy can also be obtained in bulk quantities by dealloying? (2) if so, can the high activity of extended surfaces be achieved with nanoporous Pt-Y alloy? and (3) how about its electrocatalytic activity as anode catalysts, which has not been discussed previously. Considering the limited reports in this new emerging field, further exploration is really deserved.

In this work, we successfully synthesized nanoporous Pt-Y (NP-PtY) alloy by a simple chemical dealloying process from PtYAl ternary alloy. The resulting NP-PtY alloys show bicontinuous nanoporous structure with ligaments of ~5 nm. Due to the intrinsic superior electrocatalytic activity for Pt-Y alloy combined with the advantages of 3D nanoporous structure, the prepared NP-PtY catalyst exhibit enhanced electrocatalytic performances toward ORR as well as ethanol oxidation reaction (EOR) in comparison with commercial Pt/C and nanoporous Pt (NP-Pt).

## Results and Discussion

### Characterization of NP-PtY

In comparison to the bottom-up wet chemistry method, the dealloying process provides a convenient way prepare the nanoporous alloys with better composition control and high yield. Here, we selected Pt_15_Y_5_Al_80_ ternary alloy as the source precursor, because Al can be easily dissolved in a common alkaline solution without any loss of Pt and Y. The ratio of Pt to Y was selected as 3:1 since Pt_3_Y has been proved to be a good ORR catalyst both theoretically and experimentally^26^,^31^,^32^,^33^,^34^. The as-made Pt_15_Y_5_Al_80_ alloy powders were examined by scanning eletronic microscopy (SEM) and energy-dispersive X-ray spectrometer (EDS). The SEM image (Figure S1(a), Supporting information) shows that the obtained powders are composed of fine particles of 0.5~4 μm in size. The atomic composition of the precursor, determined from EDS analysis (Figure S1(b)), is in good agreement with the our initial designed ratio. After dealloying in 5 M NaOH solution for 48 h at 50 °C, EDS results (Figure S2) show that most Al has been removed from the precursor, and the bimetallic ratio between Pt and Y is around 3.4:1, which is close to feeding ratio of 3:1.

**Figure 1:**
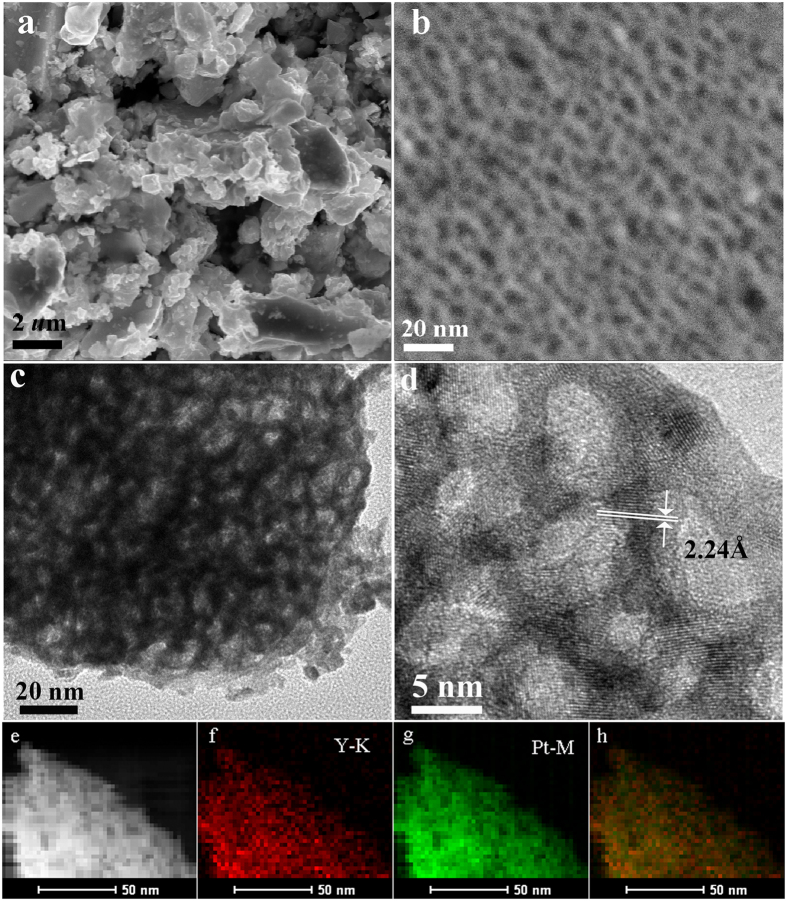
SEM (**a,b**), TEM (**c**), HRTEM (**d**), HAADF-STEM (**e**) and the corresponding elemental mapping of Pt (**g**), Y (**f**) and the overlay of Pt and Y (**h**) images of the resulted sample by dealloying of PtYAl alloy in 5 M NaOH solution for 48 h at 50 °C.

The microstructure of the dealloyed sample was characterized by electronic microscope. The SEM image in Fig. 1(a) shows that the dealloyed powders retain the particle shape of precursor, while a nanoporous morphology inside the particles can be clearly observed in Fig. 1(b). TEM image further confirms the bicontinuous three-dimensional nanoporous structure (Fig. 1(c)), which demonstrates a uniform distribution of dark metallic ligaments and bright pores. Figure 1(d) shows the high-resolution TEM (HRTEM) image of the dealloyed sample, from which a highly ordered continuous fringe pattern clearly resolved along one ligament. From the HRTEM image shown in Fig. 1(d), a highly ordered continuous lattice fringe pattern can been seen across the whole ligaments, indicating the good crystalline nature of the ligaments with average size of ~5 nm. The lattice space was calculated to be ~0.224 nm, corresponding to the (111) crystal plane of the PtY alloy. Figure 1(e)–(h) show high-angle annular-dark-field scanning transmission electron microscope (HAADF-STEM) image and STEM-energy dispersive X-ray (STEM-EDX) elemental mapping images, demonstrating the uniform distribution of Y element in Pt matrix which implies the formation of the PtY alloy. Thus, it is evident that after the dissolution of Al from the PtYAl precursor, the remaining Pt and Y atoms can form a 3D bicontinuous nanoporous structure.

**Figure 2:**
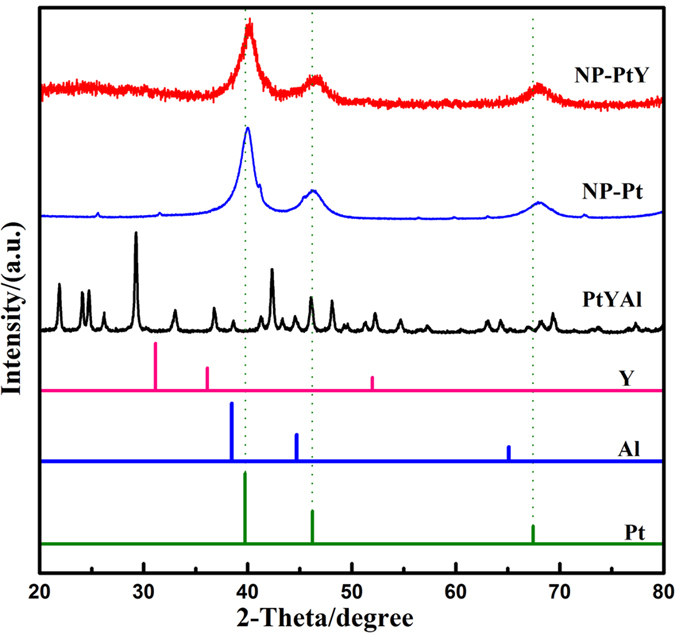
XRD patterns of PtYAl, NP-PtY, and NP-Pt. The standard patterns of pure Al (JCPDS 65–2869), Y (JCPDS 88–2328), Pt (JCPDS 65–2868) are attached for comparison.

Figure 2 shows the X-ray diffraction (XRD) patterns of the PtYAl precursor NP-PtY, and NP-Pt. For comparison, the standard patterns of pure Pt, Y, and Al metals are also included. For the PtYAl precursor, no peaks of pure Pt and Y metals can be observed, indicating the formation of PtYAl alloys. The complicated pattern of mother alloy make it difficult to index by the alloys of Pt, Y, and Al. Nevertheless, similar to the patterns of NP-Pt, after selective leaching of Al from the ternary alloy, the peaks of the PtYAl precursor disappeared, accompanied by the formation of a single phase PtY alloy. There are only three broad diffraction peaks observed with 2θ values of 40.20, 46.68, and 68.08^o^, which can be assigned to the (111), (200), and (220) diffractions of a face centered cubic (fcc) PtY alloy structure. We noted that this structure is different from that of Pt_3_Y intermetallics with primitive cubic structure. Based on the XRD patterns, the lattice constant (a) of NP-PtY is determined to be 3.880 Å, ~1.1% and 0.5% smaller than that of pure Pt (3.924 Å) and NP-Pt (3.898 Å), respectively. This results indicate the contraction of Pt lattice by Y doping, i.e., a compressive strain versus Pt. The calculated nearest Pt-Pt distance, i.e. 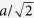, is about 2.744 Å, which is close to the value of Pt_78_Y_22_ (~2.735 Å) obtained from Fig. 3 of ref. 28 and smaller than that of pure Pt (2.775 Å). This result suggests a certain amount of Y remaining in the dealloyed nanoporous sample. Considering the larger covalent radius of Y than Pt^49^, one possible reason for the contraction of lattice is that the electronic density of Y is affected by Pt due to its high electron affinity resulting in a decrease in the radius of Y atom, as propsed in sputter-deposited Pt-Y films^32^. Hernandez-Fernandez *et al*. also observed the decrease of nearest Pt-Pt distance in Pt-Y nanoparticles in comparison to pure Pt, and explained it in terms of the formation of Pt_5_Y phases^35^. Furthermore, the broad diffraction peak in NP-PtY may arise from small grain size and large microstrains formed during dealloying process.

**Figure 3:**
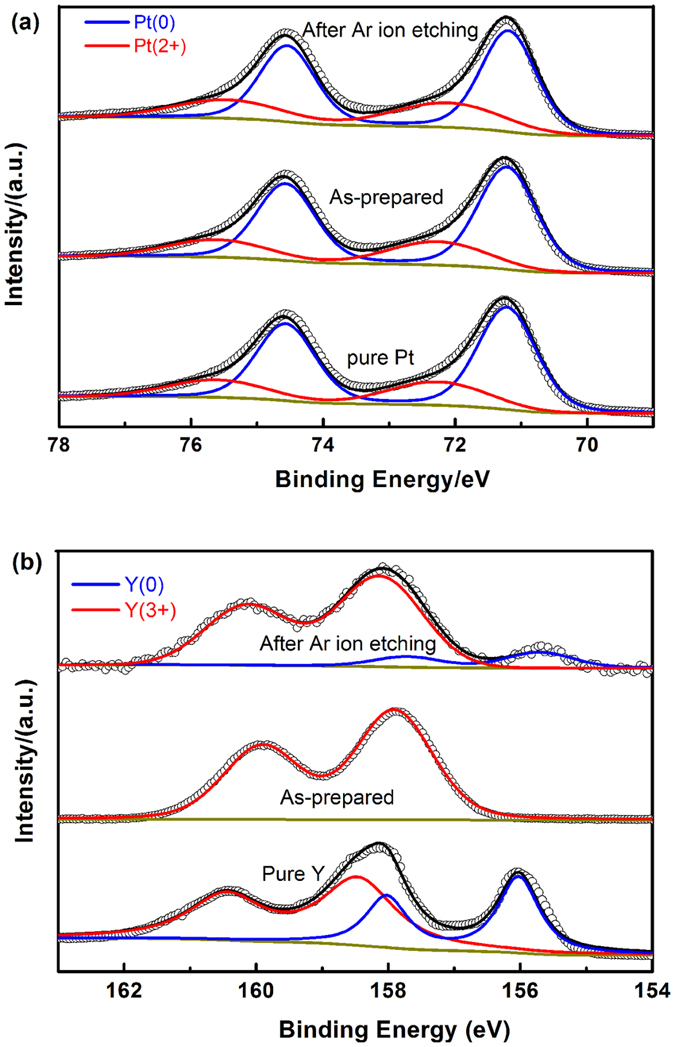
XPS spectra of (**a**) Pt 4f and (**b**) Y 3d core levels for the as prepared and Ar-ion etched NP-PtY alloy. For comparison, the spectra of pure Pt and pure Y are also attacted.

The surface electron structure of the as-prepared NP-PtY sample was examined by X-ray photoelectron spectroscopy (XPS). Figure 3a and b show the Pt 4f and Y 3d core level spectra of as-prepared and Ar-ion etched NP-PtY. For comparison, the spectra of pure Pt and pure Y are also attacted. As shown in Fig. 3(a), for as-prepared NP-PtY, the major doublet at 71.2 and 74.6 eV can be assigned to Pt 4f_7/2_ and 4f_5/2_ peaks of metallic Pt (0)^50^, almost identical to those of pure Pt; the small doublet at 72.1 and 75.5 eV originates from Pt (2+)^51^. The Y 3d core level spectra for NP-PtY (Fig. 3(b)) was split into two peaks at 157.9 and 159.9 eV, which is consistent with 3d_5/2_ and 3d_3/2_ spin doublets of Y_2_O_3_^35^. No obvious peaks of metallic Y were observed, indicating that Y oxides were the predominant species on the NP-PtY alloy surface, which is due to the oxidation of Y atoms on the nanoporous surface during dealloying and drying. From XPS results, the bimetallic ratio between Pt and Y on the surface is determined to be about 2:1, somwhat larger than feeding ratio of 3:1, which confirms the growth of Y oxide on the surface during the dealloying process. To confirm the formation of Pt-Y alloy in NP-PtY, we performed the XPS analysis on the sample after Ar ion etching. We note that the Pt 4f spectra also consists of major Pt (0) doublet and minor Pt (II) doublet, while for the spectra of Y 3d, a minor metallic Y doublet at 155.7 and 157.7 eV appears, close to those of pure Y (156.2 and 158.2 eV), suggesting the formation of Pt-Y alloy inside NP-PtY^35^. Thus, it can be deduced that, during the Al dissolution process, Y atoms at the ligament surface tend to form the Y oxides, while Y atoms inside the ligament forms Pt-Y alloy due to exceptionally negative value of Pt-Y alloy formation energy^26^.

### Electrocatalytic activities of NP-PtY towards ORR

**Figure 4:**
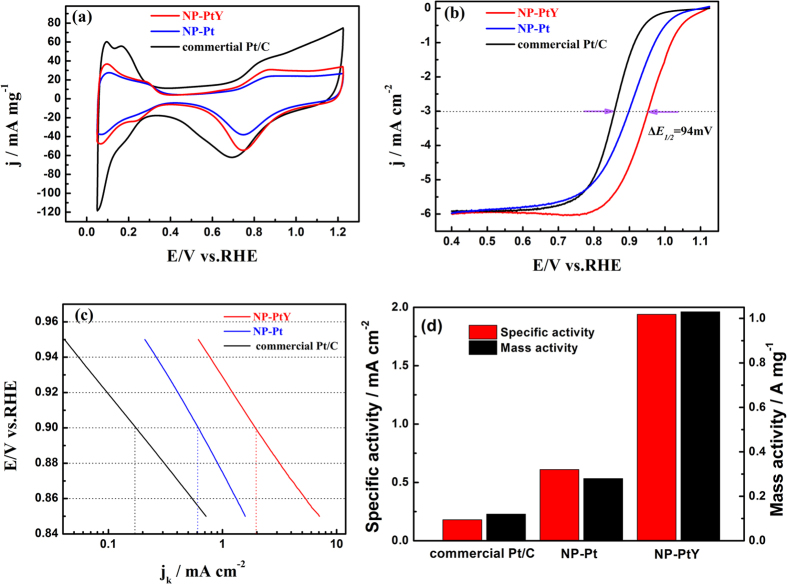
(**a**) CV curves recorded at room temperature in a N_2_-purged 0.1 M HClO_4_ solution (scan rate: 50 mV s^−1^). (**b**) Corresponding ORR polarization curves recorded in an O_2_-saturated 0.1 M HClO_4_ solution at 1600 rpm (scan rate: 10 mV s^−1^). (**c**) Specific kinetic current densities (j_K_) at different potentials. (**d**) The specific and mass activities for all catalysts at 0.9 V (vs. RHE).

Figure 4(a) shows the cyclic voltammograms (CVs) of NP-PtY and NP-Pt in N_2_-saturated 0.1 M HClO_4_ along with the commercial Pt/C catalysts for comparison. The CVs of NP-PtY and NP-Pt exhibit a similar voltammetric behaviors to that of the Pt/C catalyst. The common hydrogen underpotential adsorption/desorption (H-UPD) appears in the potential range of 0.05–0.4 V, the double-layer capacitance region locates from 0.4 to 0.6 V, and Pt oxidation/reduction peaks are in the range of 0.6−1.2 V. The absence of Y signal in the CV curve of NP-PtY indicates the formation of a pure Pt surface due to the dissolution of less-noble metal after dealloying^52^. Similar structure has also been observed in some other dealloyed nanoporous PtM (M = Cu, Ni, Co, etc) alloys, where the chemical state of Pt overlayer is influenced by the underlying PtM core, demonstrating enhanced ORR activity^46^,^48^,^53^,^54^. Compared with the commercial Pt/C catalyst, the NP-PtY and NP-Pt catalyst show a slight positive shift of the reduction peak. This positive shift suggests the weakening of Pt-oxygenated species, a sign of enhanced ORR performance. The electrochemical active surface area (ECSA) of the NP-PtY and NP-Pt, determined from the charge of hydrogen adsorption, are ~53 m^2^ g^−1^_Pt_. and ~46 m^2^ g^−1^_Pt_., respectively, which is comparable to that of the commercial Pt/C of ~68 m^2^ g^−1^_Pt_.

ORR measurements were performed in O_2_-saturated 0.1 M HClO_4_ solution at 10 mV/s at room temperature. Figure 4(b) shows the ORR polarization curves for the NP-PtY, NP-Pt, and commercial Pt/C catalysts. For all of these catalysts, the well defined diffusion controlled region was observed between 0.4 and 0.8 V with a diffusion limited current density of ~6 mA/cm^2^, and the mixed kinetic−diffusion control region occurs between 0.8 and 1.12 V. Compared to commercial Pt/C (0.859 V), NP-PtY and NP-Pt alloys exhibit much higher half-wave potentials (E_1/2_) of 0.953 and 0.897 V, respectively, which show significant positive shifts of 94 mV and 38 mV, demonstrating that both NP-PtY and NP-Pt have better electrocatalytic activity toward ORR than commercial Pt/C. To evaluate the intrinsic activity of the electrocatalysts for ORR, the ECSA specific kinetic current density (*j*_*k*_) was calculated using the Koutecky–Levich equation. As observed in the Tafel plots for *j*_*k*_ in Fig. 4(c), both NP-PtY and NP-Pt exhibit notably improved *j*_*k*_ than that of commercial Pt/C catalyst in the potential range of 0.85–0.95 V. Figure 4(d) shows the specific and mass activities calculated by normalizing the kinetic currents at 0.9 V to the ECSA and Pt mass. The specific kinetic current of NP-PtY is 1.94 mA cm^−2^ Pt at 0.9 V, which is 10.8 and 3.2 times higher than that of the Pt/C catalyst (0.18 mA cm^−2^) and NP-Pt (0.61 mA cm^−2^ Pt). In addition, the mass activity of the NP-PtY (1.03 A mg^−1^) is about 8.6 times of that for Pt/C (0.12 A mg^−1^), and 3.7 times of that for NP-Pt (0.28 A mg^−1^). The origin of the remarkably enhanced ORR performance of the NP-PtY catalyst should be considered by combining the intrinsic catalytic property of Pt_3_Y and the special nanoporous structure. First, a compressive strain in NP-PtY compared to pure Pt, as demonstrated in XRD results, would increase the width of Pt d band, leading to the downshift of d-band center and weakening the binding strength to the O-containing reaction intermediates^55^,^56^. Second, the large difference in electronegativity between Pt and Y causes the electron transfer from Y to Pt and result in higher d-band filling^34^,^57^. This may also lower the d-band center. In addition, the 3D bicontinuous nanoporous structure in NP-PtY facilitates electron conductivity for Pt surface sites, which is favorable for reaction kinetics on the catalyst surface.

**Figure 5:**
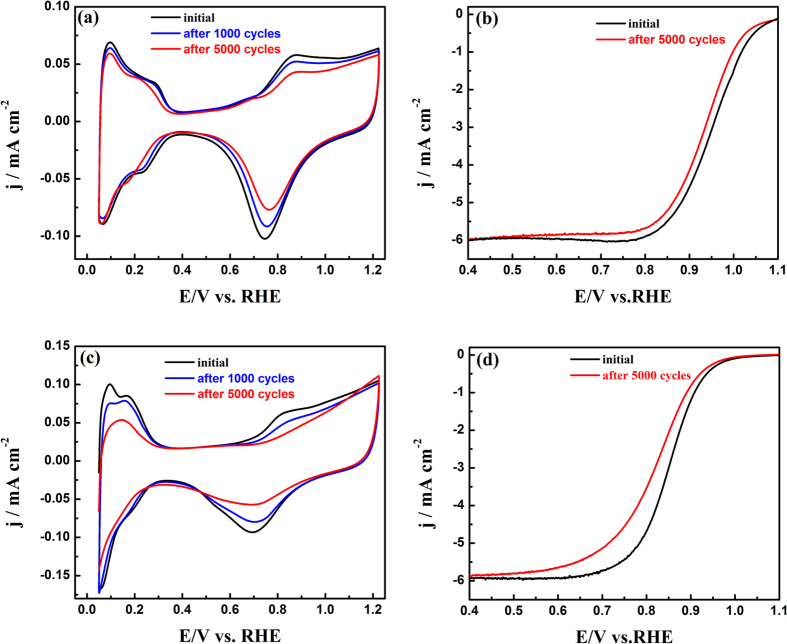
Cyclic voltammograms for (**a**) NP-PtY and (**c**) Pt/C catalysts before and after ADT at a scan rate of 50 mV s^−1^, respectively. ORR polarization curves for (**b**) NP-PtY and (**d**) Pt/C before and after ADT.

To evaluate the stability of the NP-PtY, the accelerated durability tests (ADT) of electrocatalysts were conducted by continuously cycling the potential between 0.6 and 1.2 V (vs. RHE) in O_2_-saturated 0.1 M HClO_4_ solution at a scan rate of 100 mV s^−1^, based on an established procedure^58^,^59^. As shown in Fig. 5(b), NP-PtY showed a slight loss of 9.3% in ECSA after 1000 cycles, while the commercial Pt/C showed a significant loss of 24%. After 5000 cycles, the CV measurements showed a spot of drop of only 18.1% in ECSA for NP-PtY, but a substantial loss of 60.7% for Pt/C shown in Fig. 5(d), suggesting that the NP-PtY had a better durability than the Pt/C catalysts. Furthermore, ORR polarization curves were also measured at 5000 cycle intervals. After 5000 cycles, there is only a 15 mV degradation in the half-wave potential E_1/2_ for the NP-PtY (Fig. 5(a)), while the corresponding potential E_1/2_ for Pt/C decreases by 27 mV (Fig. 5(c)). The ADT results demonstrate that the NP-PtY have remarkably higher stability compared to commercial Pt/C. The enhanced durability can be partly attributed to the negative heat of formation in alloy of Pt with Y, which provides them higher kinetic stability against continuous dealloying under fuel cell reaction conditions than alloys of Pt and late transition metals. The EDS result (Figure S3(a)) after ADT demostrates a small dissolution of Y and Al during ADT, and the Pt/Y ratio is around 5.1:1. The 3D nanoporous structure of NP-PtY can effectively overcome the common problems of nanoparticle aggregation and loss of electric contact to carbon support observed in the particle-type electrocatalysts such as comercial Pt/C. The small decrease of ECSA in NP-PtY should be due to the coarsening of the interconnected ligaments in the NP architecture according to the power law decay^60^. This character was demonstrated in TEM image of NP-PtY after ADT (Figure S3(b)), which shows that the size of ligaments after ADT grows to about 7 nm from 5 nm in original NP-PtY.

### Electrocatalytic activity and stability for EOR

**Figure 6:**
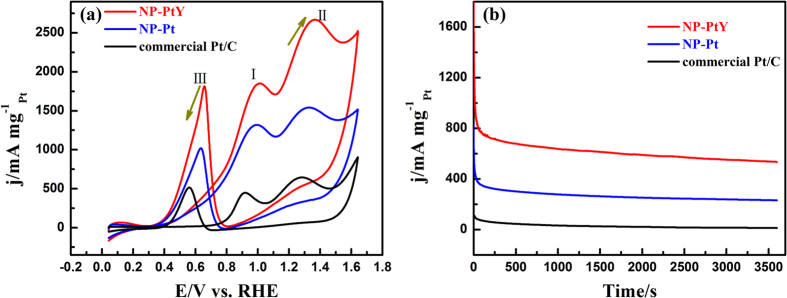
(**a**) Cyclic voltammograms and (**b**) chronoamperometric curves of NP-PtY, NP-Pt and commercial Pt/C catalyst-modified electrodes in 0.5 M H_2_SO_4_ + 1 M ethanol under N_2_ atmosphere normalized by the Pt mass; scan rate: 50 mV s^−1^.

To further investigate the electrocatalytic performance of the as-prepared catalysts, EOR was investigated on NP-PtY catalyst-modified electrodes. For comparison, NP-Pt and the commercial Pt/C were also characterized under the same experimental conditions. Figure 6(a) shows the corresponding CV curves in a N_2_-saturated 0.5 M H_2_SO_4_ containing 1 M ethanol at a scan rate of 50 mV s^−1^. In all of the CV curves, three anodic peaks were observed in the positive scan and the reversed negative scan, which are well-known to reflect the electrooxidation of ethanol and intermediate carbonaceous species (e.g., CH_3_CHO), respectively. Accordingly, the current density of the forward oxidation peak (I, ca.0.95 V) can be employed to evaluate the catalytic activity of a catalyst. As can be seen from Fig. 6a that the mass activities of NP-PtY (1.83 A mg^−1^) is 1.4 and 4.1 times higher than that of NP-Pt (1.31 A mg^−1^) and commercial Pt/C (0.45 A mg^−1^). The onset potential is a key parameter for evaluating the activity of catalyst. The onset potential of the NP-PtY electrode is 0.54 V, much lower than that of the commercial Pt/C catalyst (0.72 V). The shift of the onset potential to a more negative value indicates that NP-PtY can enhance the electro-oxidation kinetics of ethanol, which is important for the application in fuel cells. The lower onset potential and higher mass activity of the NP-PtY for catalyst ethanol oxidation indicate that it is superior to the other catalysts.

Furthermore, in order to estimate the tolerance of the catalysts, chronoamperometric measurements were performed in 0.5 M H_2_SO_4_ containing 1 M ethanol. Figure 6(b) shows the current-time curves for EOR measured at a fixed potential of 0.84 V on NP-PtY modified electrodes, using NP-Pt and the commercial Pt/C catalysts as references. During the whole reaction process, the ethanol oxidation current densities on the NP-PtY was higher than those on NP-Pt and commercial Pt/C catalysts. Meanwhile, it is observed that the current densities decrease more sluggishly for NP-PtY as compared to the other two catalysts, indicating a higher tolerance to the carbonaceous species generated during ethanol oxidation.

## Conclusions

In summary, we have successfully synthesized nanoporous Pt-Y alloy with ligaments of ~5 nm by a simple one-step dealloying process. Compared with NP-Pt and commercial Pt/C catalysts, the resulting NP-PtY catalysts displayed superior ORR and EOR activity as well as long term durability. The strain effect and electronic effect by alloying of Pt and Y may account for the improvement of electrocatalytic activity. In addition, the 3D nanoporous structure is beneficial for the electroreduction of oxygen associated with fast transportation of electrons and medium molecules through the interconnected ligaments and bi-continuous pores, respectively. Our results suggest that the dealloying process is suitable for large-scale synthesis of NP-PtY and may be used to prepare other nanoporous Pt-rare-earth alloys as ORR and EOR catalysts. The superior catalytic performance and facile preparation suggest the as-made NP-PtY holds great promise as electrocatalyst in PEMFCs.

## Methods

### Preparation of nanoporous Pt-Y and Pt

Pt_15_Y_5_Al_80_ and Pt_20_Al_80_ alloy ingots were prepared by arc melting the corresponding pure metals Pt, Y, and Al (99.99%) argon atmosphere repeatedly. The ingots were subsequently grounded to fine powders by a mortar. Nanoporous Pt-Y (NP-PtY) alloy was prepared by dealloying the Pt_15_Y_5_Al_80_ powders in N_2_-bubbled 5 M NaOH solution for 48 h at 50 °C. For comparison, NP-Pt alloy was also fabricated by dealloying Pt_20_Al_80_ in the same condition. The samples were washed several times with ultrapure water (18.2 MΩ) and dried at room temperature in air.

### Characterization

The X-ray diffraction (XRD) patterns were acquired on a Rigaku D/max 2200/PC diffractometer equipped with graphite-monochromatized Cu K_α_ radiation (λ = 1.54060 Å) in 2θ ranging from 20° to 80°. The microstructure and chemical composition were characterized by a Zeiss Sigma field emission scanning electronic microscopy (FESEM) equipped with an Oxford INCA energy-dispersive X-ray spectrometer (EDS). The transmission electron microscopy (TEM) images were taken on a Tecnai G220S-TWIN (FEI, America) with an accelerating voltage of 200 kV. The electron structures of NP-PtY were analyzed with a Thermo ESCALab250 X-ray photoelectron spectrometer (XPS) using monochromatized Al Ka radiation.

### Electrochemical measurement

The electrochemical measurements were carried out on a CHI 760E electrochemical workstation (CH Instruments, Inc.) using conventional three electrode system: a modified rotating disk glassy carbon electrode (RDE, diameter = 5 mm; area = 0.196 cm^2^) or glassy carbon electrode (GCE, diameter = 3 mm; area = 0.071 cm^2^) as the working electrode, a platinum wire served as the counter electrode, and a saturated calomel electrode (SCE) or an Ag/AgCl electrode (saturated KCl) used as the reference electrode. Prior to the start of the electrochemical study, the working electrode (RDE and GCE) were polished with 0.05 μm alumina slurry and were sonicated for a few minutes in ethanol and then in DI water.

To prepare working electrodes, 1 mg NP-PtY or NP-Pt samples and 2 mg of carbon powder (Vulcan XC-72R, Cabot) were dispersed in a mixture of water/isoproyl alcohol/Nafion (with a volume ratio of 1:4:0.025) and sonicating for 1 h to form a 3 mg/mL catalyst ink. Then 3 and 5 μL of suspension was dropped onto the clean RDE and GCE electrode. After that, it was dried in air at room temperature. For comparison, the commercial Pt/C catalyst ink (20 wt.%, John-Matthey, purchased from Alfa Aesar) was also prepared in a similar way without further addition of carbon powder. The corresponding Pt loadings on the modified RDE and GCE were determined to be 15.3 and 34 μg cm^−2^, respectively.

The cyclic voltammetry (CV) tests were performed in N_2_-purged 0.1 M HClO_4_ solution at room temperature. The electrode potential was scanned in the range from 0.05 to 1.2 V (vs. RHE) at a scan rate of 50 mV s^−1^. The specific electrochemically active surface area (ECSA) of Pt was estimated from the area of desorption of atomic hydrogen on the curve of the CV between ca.0.05 and 0.4 V by the following equation:


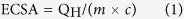


where Q_H_ is the charge for hydrogen desorption (mC cm^−2^), m is the Pt loading (mg cm^−2^) in the electrode, and c is the charge required for the monolayer adsorption of hydrogen on a Pt surface (0.21 mC cm^−2^).

The electrocatalytic activity for the ORR was conducted using a rotating disk electrode controller (AFMSRCE, Pine Instrument Co.) in an O_2_-saturated 0.1 M HClO_4_ solution by sweeping the potential from 0.4 to 1.12 V (vs. RHE) with linear scanning voltammetry (LSV) at a scan rate of 10 mV s^−1^ with a rotation rate of 1600 rpm. The kinetic currents densities (*j*_*k*_), which represent the intrinsic ORR activity of the catalysts, were calculated by the Koutecky-Levich (K-L) equation:


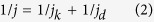


where *j* is the measured current density, *j*_k_ and *j*_d_ are the kinetics-limited and the diffusion-limited current densities, respectively. The stability test was performed by cycling the catalyst in the potential range of 0.6 V to 1.2 V at a rate of 100 mV s^−1^ for 5000 scans in O_2_-saturated 0.1 M HClO_4_.

The electrocatalytic oxidation of ethanol were performed in N_2_-saturated 0.5 M H_2_SO_4_ solution containing 1.0 M ethanol at a scan rate of 50 mV s^−1^.

## Additional Information

**How to cite this article**: Cui, R. *et al*. Facile Synthesis of Nanoporous Pt-Y alloy with Enhanced Electrocatalytic Activity and Durability. *Sci. Rep.* **7**, 41826; doi: 10.1038/srep41826 (2017).

**Publisher's note:** Springer Nature remains neutral with regard to jurisdictional claims in published maps and institutional affiliations.

## Supplementary Material

Supporting Information
