# Supplementary material for: Facile Synthesis of Nanoporous Pt-Y alloy with Enhanced Electrocatalytic Activity and Durability
Source: Sci Rep. 2017 Feb 2;7:41826. doi: 10.1038/srep41826 (PMC5288775; doi:10.1038/srep41826)
Supplement: Supporting Information [file srep41826-s1.doc]

Supporting Information

**Facile Synthesis of Nanoporous Pt-Y alloy with Enhanced Electrocatalytic Activity and Durability**

Rongjing Cui ^1^, Ling Mei ^1,2^, Guangjie Han ^1,2^, Jiyun Chen ^4,5^, Genhua Zhang ^3^, Ying Quan ^3^, Ning Gu ^2^, Lei Zhang ^4^, Yong Fang ^4^, Bin Qian ^4^, Xuefan Jiang ^4^ & Zhida Han ^4,*^

^1^ Department of Chemistry and Materials Engineering, Changshu Institute of Technology, Changshu 215500, People’s Republic of China

^2^ Department of Chemistry and Materials, Hebei Normal University, Shijiazhuang 050024, People’s Republic of China

^3^ Suzhou Key Laboratory of Food Quality and Safety, School of Biology and Food Engineering, Changshu Institute of Technology, Changshu 215500, People’s Republic of China

^4^ Jiangsu Laboratory of Advanced Functional Materials, Department of Physics, Changshu Institute of Technology, Changshu 215500, People’s Republic of China

^5^ School of Materials Science and Engineering, China University of Mining & Technology, Xuzhou 221116, People’s Republic of China

* Corresponding authors: [*han@cslg.cn*](mailto:han@cslg.cn)

**Contents**

Figure S1 (a) SEM image of the PtYAl alloy powders; (b) EDS spectra of the PtYAl alloy powders.

Figure S2 EDS spectra of the dealloyed sample (NP-PtY) in 5 M NaOH solution at 50 ^o^C for 48 h.

Figure S3 (a) EDS spectra, and (b) TEM image of NP-PtY after ADT.


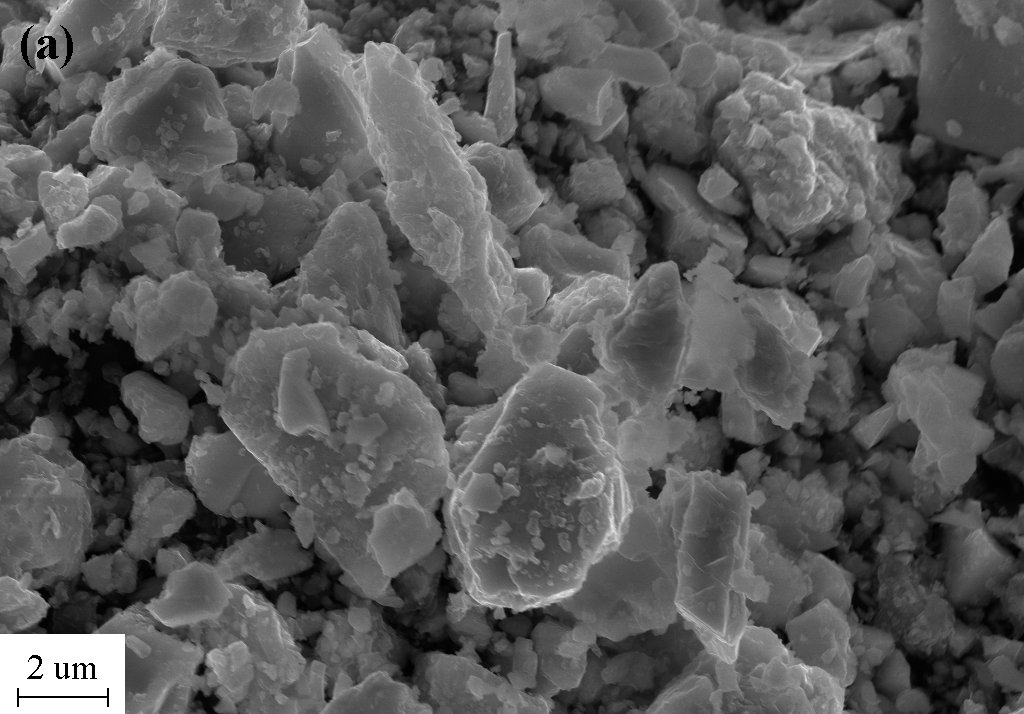


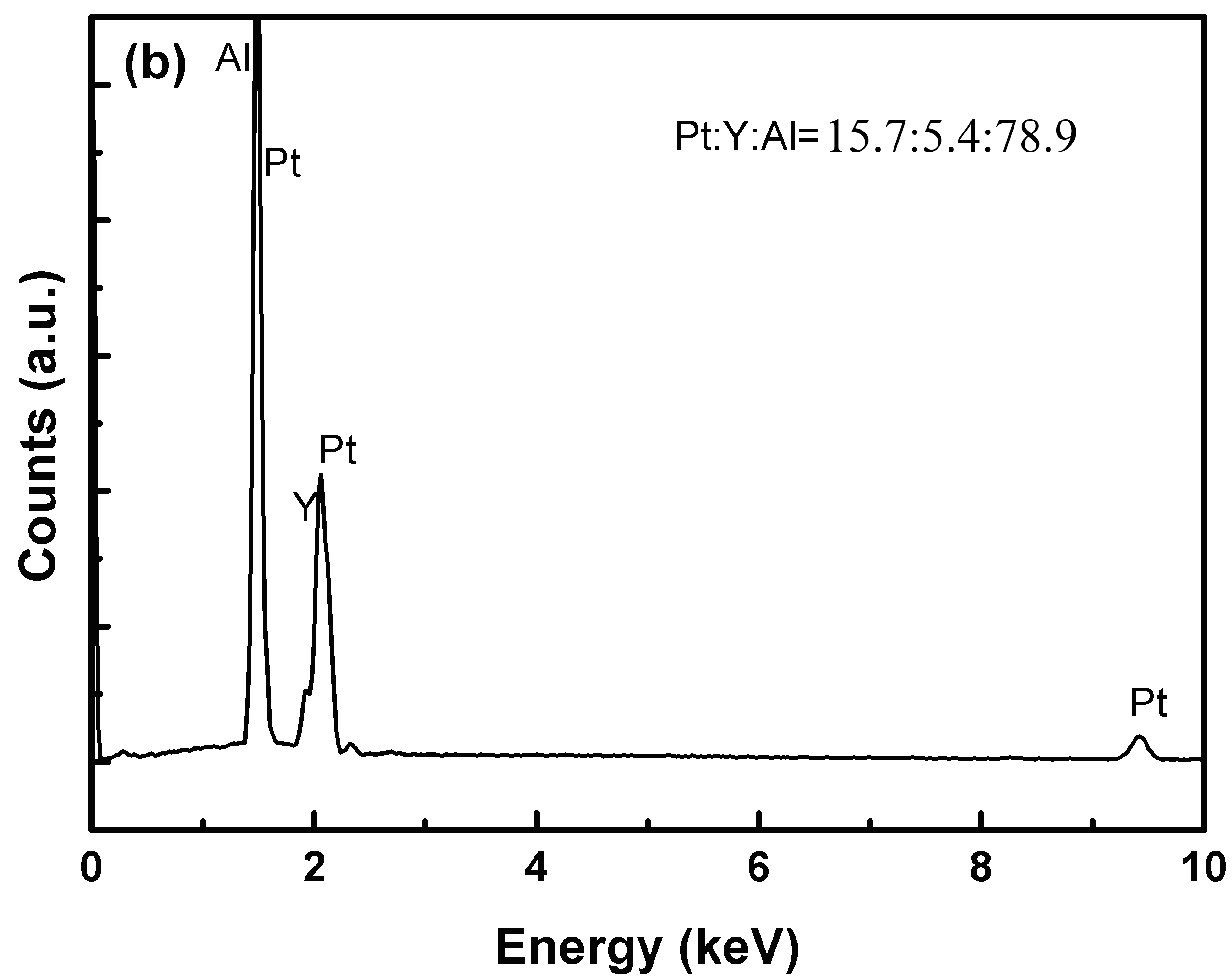


**Figure S1**. (a) SEM image of the PtYAl alloy powders; (b) EDS spectra of the PtYAl alloy powders.


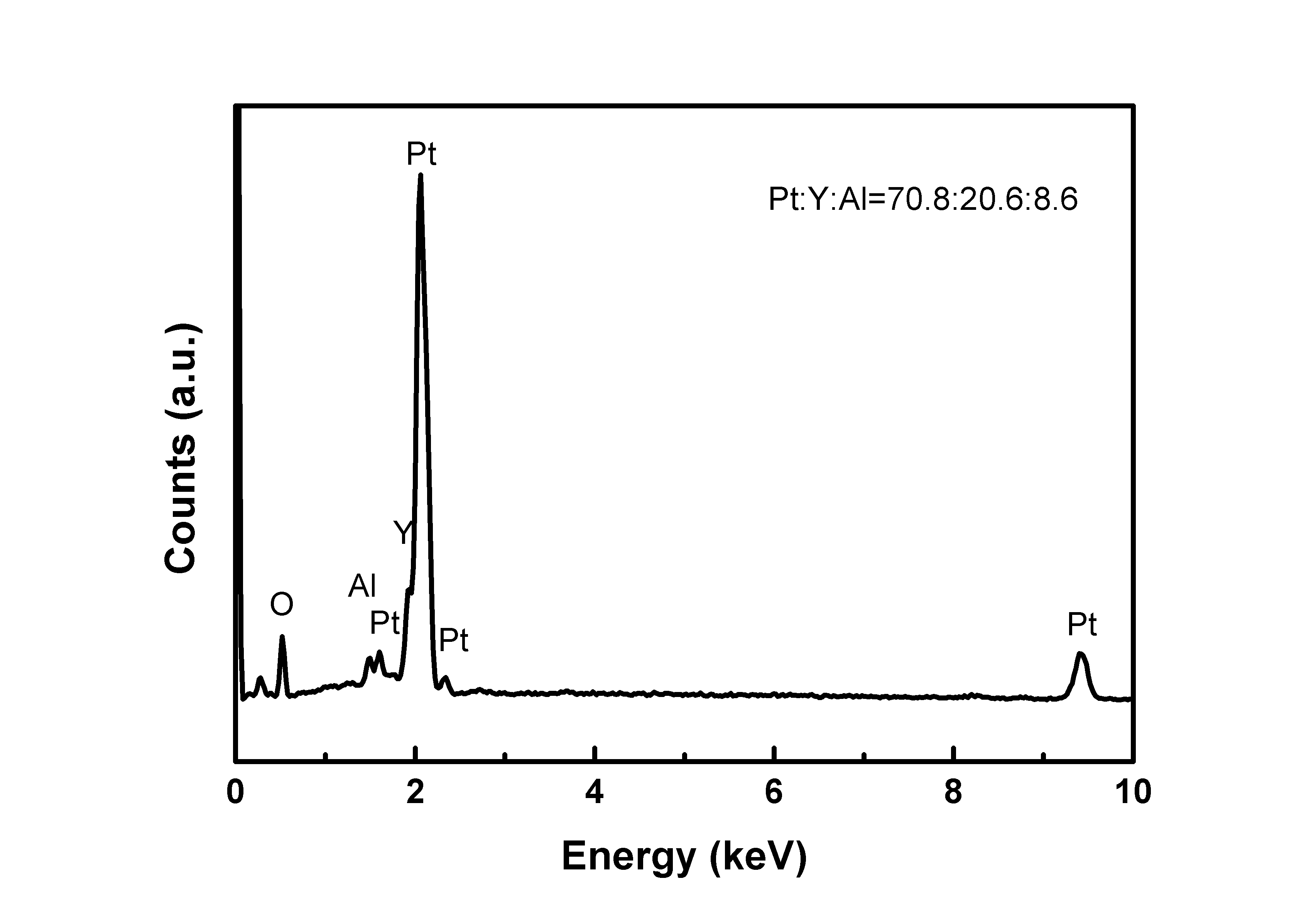


**Figure S2**. EDS spectra of the dealloyed sample (NP-PtY) in 5 M NaOH solution at 50 ^o^C for 48 h.


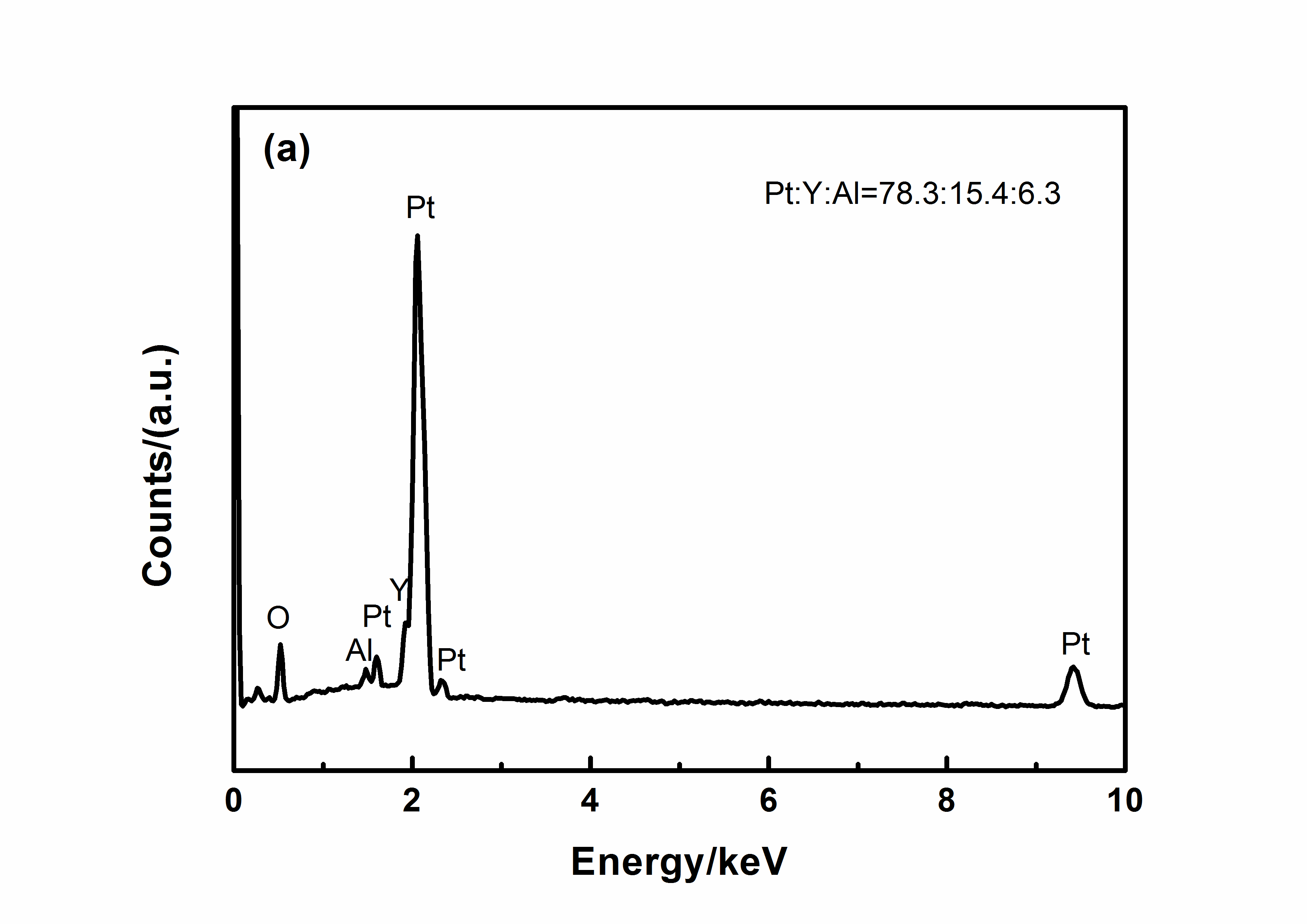


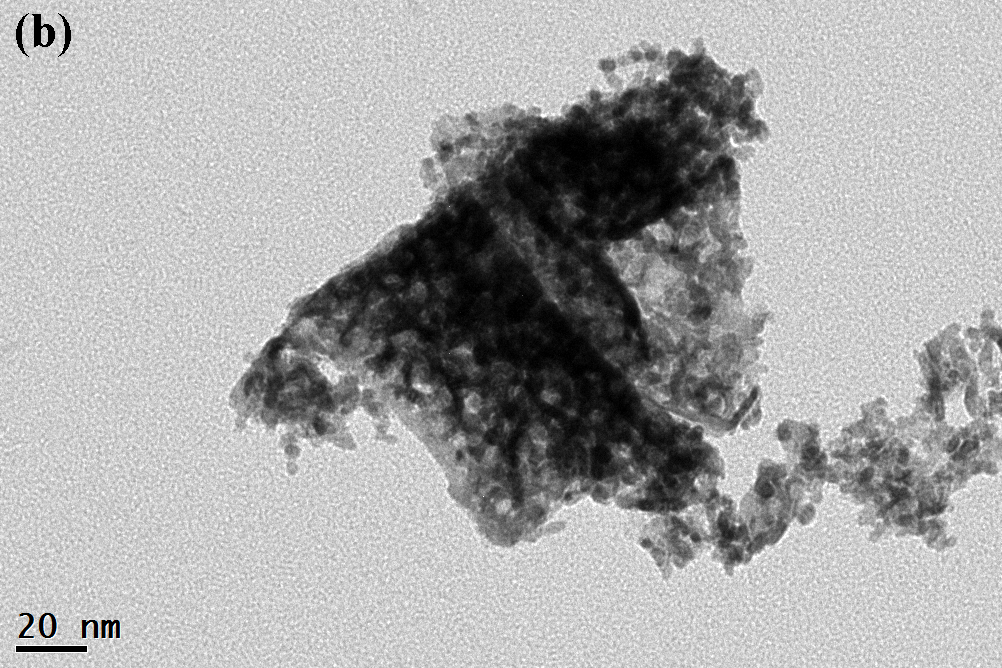


**Figure S3**. (a) EDS spectra, and (b) TEM image of NP-PtY after ADT.
